# Use of the ThinPrep^® ^Imaging System does not alter the frequency of interpreting Papanicolaou tests as atypical squamous cells of undetermined significance

**DOI:** 10.1186/1742-6413-5-10

**Published:** 2008-04-24

**Authors:** Michael J Thrall, Donna K Russell, Thomas A Bonfiglio, Rana S Hoda

**Affiliations:** 1 Department of Pathology, University of Rochester Medical Center, Rochester, New York, USA

## Abstract

**Background:**

Automated screening of Papanicolaou tests (Pap tests) improves the productivity of cytopathology laboratories. The ThinPrep^® ^Imaging System (TIS) has been widely adopted primarily for this reason for use on ThinPrep^® ^Pap tests (TPPT). However, TIS may also influence the interpretation of Pap tests, leading to changes in the frequency of various interpretive categories. The effect of the TIS on rates of TPPT interpretation as atypical squamous cells of undetermined significance (ASC-US) is of concern because any shift in the frequency of ASC-US will alter the sensitivity and specificity of the Pap test. We have sought to determine whether automated screening of TPPT has altered ASC-US rates in our institution when compared with manual screening (MS) of TPPT.

**Methods:**

A computerized search for all ASC-US with reflex Human Papillomavirus (HPV) testing over a one-year-period (7/1/06 to 6/30/07) was conducted. Cases included both TPPT screened utilizing TIS and screened manually. HPV test results for both groups were recorded. Pertinent follow-up cervical cytology and histology results were retrieved for the period extending to 11/30/07. Automated screening was in clinical use for 10 months prior to the start of the study.

**Results:**

Automated screening was performed on 23,103 TPPT, of which 977 (4.23%) were interpreted as ASC-US. Over the same period, MS was performed on 45,789 TPPT, of which 1924 (4.20%) were interpreted as ASC-US. Reflex HPV testing was positive for high risk (HR) types in 47.4% of the TIS cases and 50.2% of MS cases. Follow-up cervical dysplasia found by colposcopy was also distributed proportionally between the two groups. Cervical intraepithelial neoplasia (CIN) was found on follow-up biopsy of 20.1% of the TIS cases (5.2% CIN 2/3) and 21.2% of MS cases (5.1% CIN 2/3). None of these differences were statistically significant.

**Conclusion:**

Use of the ThinPrep^® ^Imaging System did not appreciably change ASC-US rates or follow-up reflex HPV test results in our laboratory. This demonstrates that the benefits of automated screening may be obtained without increasing the rate of referral to colposcopy for ASC-US follow-up.

## Introduction

Automated screening of ThinPrep^® ^Papanicolaou Tests (TPPT) has become increasingly common in clinical practice. The ThinPrep^® ^Imaging System (TIS) (Hologic Corp. [previously Cytyc Corp.], Marlborough, MA) is the most widely used and studied automated system currently available. The operational details of the TIS have previously been published [1]. Briefly, the TIS is an automated imaging and review system indicated for primary screening of TPPT. The System consists of three components: an image processor, a PC-based computer that runs on Windows NT (Microsoft Corp., Redmond, WA), and a review microscope with a mechanical stage and electronic dotting capability. The TIS uses algorithms to select 22 fields of view (FOV) which include the cells most likely to be dysplastic according to computer imaging characteristics. These fields are then reviewed by a cytotechnologist (CT). If all cells seen in the 22 FOV are considered normal the case is signed out as negative by the CT. Since most slides fall into this category, thereby reducing the amount of time spent on screening, the TIS greatly reduces workload and improves laboratory productivity. If any cells within the 22 FOV are considered abnormal the entire slide is manually re-screened utilizing the automated microscope. The TIS has an additional benefit: increased sensitivity. Published studies have consistently shown that this system leads to the discovery of a higher proportion of slides with dysplastic cells leading to an interpretation of either low-grade or high-grade squamous intraepithelial lesion (LSIL or HSIL) [2-8]. This increase in sensitivity does not appear to come at the expense of specificity, as follow-up biopsies show a corresponding increase in the frequency of discovery of dysplastic lesions [3].

The effect of the TIS on rates of atypical squamous cells of undetermined significance (ASC-US), however, is less clear. Two studies reported an increase in the ASC-US rate [7,8], while two others reported a decrease [5,6] attributable to the TIS. These contrasting results occurred despite use of a similar study design that compared the ASC-US rate during the one-year periods before and after the introduction of the TIS into their respective laboratories [5-8].

ASC-US is the most common abnormal interpretation of Pap tests. Consequently, this category accounts for the highest proportion of the cervical intraepithelial neoplasia (CIN) 2 and 3 detected by screening (38.8% of cases in the study by Kinney and coworkers [9]). ASC-US rates also heavily influence the cost of cervical cancer screening programs. Current recommendations advise reflex HPV testing for ASC-US found in women over age 20 years, with subsequent colposcopic examination in women positive for high-risk (HR) types [10]. Given these facts, any effect of the TIS on the frequency of ASC-US interpretations could have significant clinical and public health implications.

If the ASC-US rate were to increase as a result of TIS use, the sensitivity of the Pap tests would almost certainly rise. However, the cost of cervical cancer screening programs would also rise. At the very least, the number of reflex HPV tests would increase. Referrals to colposcopy could also increase if the number of reflex HPV tests positive for HR-types went up as a result of more frequent testing. Depending on the size of these increases, the rise in costs could become substantial.

A decrease in the ASC-US rate as a result of using the TIS would lead to a regrettable loss of Pap test sensitivity. If the frequency of LSIL and HSIL interpretations increased simultaneously, however, this could at least partially offset the sensitivity losses. The costs of Pap test screening programs would reflect the overall sensitivity of the test, with declining ASC-US rates probably resulting in a less costly system.

The introduction of reflex HPV testing for ASC-US into clinical practice has created a new metric for the evaluation of ASC-US rates: the frequency of results positive for high-risk viral types. The ALTS trial, which established the efficacy of HPV triage, also set a benchmark for HR-HPV positive rates in ASC-US of 50% [11]. At present there is considerable variation in HR-HPV rates between laboratories [12] and between individual cytologists [13,14]. However, some academic laboratories have already established an average HR-HPV positive rate similar to that of the ALTS trial [13,14]. Many cytologists are interested in using this metric to try to achieve more standardization of the ASC-US category. Ultimately, it is hoped, this may enable the cytology community to create reproducible sensitivity and specificity characteristics for the Pap test.

This study evaluates the effect of the TIS on ASC-US rates on TPPT and subsequent follow-up in a screening population arbitrarily divided between manual and TIS-assisted screening, over a single time period, with reflex HPV test result feedback.

## Methods

Our laboratory processes approximately 70,000 Pap tests per year, of which more than 95% are TPPT with the remainder consisting of conventional smears and SurePath^® ^liquid-based preparations (BD, Franklin Lakes, MD). This study focuses on TPPT. We receive specimens from a mix of clinical settings which include both low-risk populations (privately insured women) and high-risk populations (prisoners and recipients of subsidized women's health services). Specimens were examined by a team of 12 CTs and 4 cytopathologists during the study period. The ASC:SIL ratio for the laboratory was 1.3 in 2006 and 2007.

The TIS has been in clinical use in our laboratory since September of 2005. Although all TPPT were processed through the imager during the study period, many were manually screened because of an insufficient number of special automated microscopes. Selection for MS was made essentially randomly by the CT with no systematic bias we are aware of. Cases screened with the assistance of the TIS are identified by a standardized comment included in the reports of all such cases.

Reflex HPV testing is performed by the Hybrid Capture 2 method (Digene, Gaithersburg, MD) using a mixed probe targeting 13 cancer-associated HR-HPV types. The test is performed by the microbiology section of our department based on the manufacturer's protocol. Specimens without sufficient material remaining in the vial, approximately 4 mL, were excluded from this study.

The cytopathologists in our laboratory were aware of the results of reflex HPV testing for their ASC-US cases. The goal of the laboratory was to maintain a HR-HPV positivity rate of close to 50% during this period.

Cervical biopsy specimens are reviewed by the surgical pathology department which includes 19 pathologists.

The computerized laboratory records for our institution were reviewed for the one-year period from July 1, 2006 to June 30, 2007 to find all TPPT interpreted as ASC-US. Follow-up reflex HPV test and biopsy results, as well as additional Pap test results for the time period extending from July 1, 2006 to November 30, 2007, were also reviewed. The institutional review board authorized this study.

The statistical calculations employed the Yates correction of the chi-square test using an online resource [15]. We considered findings to be statistically significant at p-values < 0.05.

## Results

During the study period, 68,898 Pap tests were performed in our laboratory. Of these, 2,901 tests from 2,716 women (age range: 15–85 years; mean age: 34 years; median age: 30 years), were interpreted as ASC-US and had a reflex HPV test.

One third (33.5%) of the cases were screened with the assistance of the TIS. The comparative rates of ASC-US interpretations for the two groups as well as the reflex HPV test results are displayed in Table 1. The difference in the ASC-US rates is miniscule (4.23% for TIS cases versus 4.20% for MS) and not statistically significant (p = 0.888). HR-HPV positive rates in ASC-US are also very similar in the two groups (47.4% for the TIS versus 50.2% for MS), again without statistical significance (p = 0.383).

**Table 1:** Comparison of the automated and manual screening methods

Method	Total Tests	ASC-US		HR-HPV Positive		
		Number	% of Total	Number	% of ASC-US	% of Total
TIS	23103	977	4.23%	463	47.4%	2.00%
MS	45789	1924	4.20%	965	50.2%	2.11%

The distribution of ASC-US cases screened by the ThinPrep^® ^Imaging System (TIS) and by manual screening (MS) alone with the follow-up reflex HPV results.

Among HR-HPV positive cases, follow-up biopsy results were available for almost half of the women as shown in Table 2. Just over 5% of the HR-HPV positive women in both groups (5.18% for the TIS and 5.07% for MS; p = 0.920) had CIN 2 or 3 found by histological sampling during this short follow-up period. In addition, three cases of adenocarcinoma in-situ were found in follow-up of these women, two in the TIS group (including one in an adolescent) and one in the MS group.

**Table 2:** Biopsy results for the high-risk HPV positive subset

Method	Total ASC-US	Biopsies	% Biopsied	CIN 1		CIN 2/3	
				Number	% of Total	Number	% of Total
TIS	463	209	45.1%	69	14.9%	24	5.18%
MS	965	435	45.1%	156	16.2%	49	5.07%

Follow-up biopsy results for the ASC-US cases screened by the TIS and by MS alone among women positive for HR-HPV.

Among women negative for HR-HPV, 80 had histological follow-up, including 25 women screened by the TIS and 55 by MS. Among these there were 10 cases of dysplasia (CIN 1, 8; CIN 2, 2) in the TIS group and 11 cases (CIN 1, 10 and CIN 3, 1) in the MS group.

In the subset of HR-HPV positive women who did not have histological sampling, 387 had follow-up Pap tests available, including 132 women screened by the TIS and 255 by MS. Of these a few had an interpretation of dysplasia in both the TIS group (LSIL, 23; HSIL, 1) and the MS group (LSIL, 55; HSIL, 1).

Since reflex HPV testing in adolescent women is now deemed "unacceptable" according to the recently revised ASCCP guidelines [10] we have re-analyzed our data to gauge the effect of removing them from the reflex-tested population. The data for only those women aged more than 20 years is presented in Table 3. Eliminating reflex HPV tests for adolescents would have reduced our HPV test rate by 11.7% in the TIS group and 14.4% in the MS group (p = 0.042). HR-HPV positive result rates would have remained comparable for both groups (44.8% for the TIS versus 46.0% for MS; p = 0.572).

**Table 3:** Comparison of the automated and manual screening methods in women over age 20

Method	ASC-US	HR-HPV Positive			
		Total +	Biopsy	CIN 1	CIN 2/3
TIS	863	387	180	60	22
MS	1647	758	366	129	42

The distribution of ASC-US cases with follow-up HPV testing and biopsy results for women aged more than 20 years and screened by the TIS or by MS alone.

## Discussion

Previous studies of the clinical performance of the TIS have yielded conflicting results about the effect on the ASC-US rate. Most studies have used a non-synchronous design, with the Pap test interpretations from the year before the introduction of the TIS compared with the interpretations from the first year of TIS use. Using this method, Lozano found an increase in the ASC-US rate from 4.09% to 6.52% (p < 0.001) [7] and Dziura and coworkers found an increase from 3.1% to 4.0% (p-value not given) [6] after the introduction of the TIS to their laboratories. Both demonstrated a corresponding decrease in the frequency of detection of HR-HPV on reflex testing. Conversely, Miller and coworkers found a decrease in the ASC-US rate from 5.59% to 4.72% (p < 0.0001) associated with an increase in the HR-HPV positive rate [8]. Chivukula and coworkers also found a decrease in the ASC-US rate, from 8.79% to 8.70% (p-value not given), though they did not report the follow-up HPV test results [5].

These studies, in contrast to ours, have the strength of allowing for and measuring changes in clinical practice following the introduction of the TIS. If the TIS somehow revolutionized the interpretation of Pap tests, these studies would be more likely to detect the difference. However, this design of comparing consecutive years also has serious problems. Any change in clinical practice from year to year will affect the results, confounding the analysis of the influence of the TIS alone.

Lozano demonstrated an impressive "learning curve" effect in the early months of TIS use which corresponded to a sizeable, sudden change in both the ASC-US rate and the follow-up HPV results [7]. This "learning curve" could potentially have influenced the outcomes of all four studies. Significantly, the ASC-US rate remained higher and the reflex HR-HPV rate lower in the final month of the study than in the previous year. This would indicate that the "learning curve" alone was not responsible for the entirety of the observed changes in the ASC-US rate in the Lozano study and, therefore, probably does not account for all of the effects on ASC-US rates reported in the other year-to-year comparison studies.

Another factor to consider is the influence of changing interpretive thresholds. Although it is not feasible to speculate on the cause of such changes, the consistent observation that the HR-HPV positivity rate changed in accord with the ASC-US rate in all three studies makes this mechanism a likely explanation for the findings. Lowered thresholds for ASC-US will increase the ASC-US rate and decrease the HR-HPV frequency in reflex testing, with raised ASC-US thresholds having the opposite effect. These scenarios correspond to the findings of the three studies that reported follow-up HPV results [6-8]. Of course, thresholds for LSIL also influence the HPV follow-up of ASC-US, but these three studies reported similar 37–46% increases in the LSIL rates from one year to another [6-8]. Therefore changes in LSIL thresholds are unlikely to account for their very different HPV testing results.

Our study, in comparison with these, has the merit of synchronous analysis of the TIS and MS groups. This should minimize interpretive threshold differences and other potentially confounding factors such as changes in cytology staffing or differing specimen sources over time. We also designed our study to begin well after the introduction of the TIS to our laboratory (10 months) to eliminate any potential "learning curve" effect.

Only a few studies have analyzed the effects of the TIS on ASC-US rates using single-patient comparisons [3,4]. Unfortunately, these shed little light on the question at hand. Biscotti and coworkers screened the same slides manually and with the assistance of the TIS [3]. They found an increase in the ASC-US rate in the TIS subset upon initial review, but a decrease in the same subset following adjudication by multiple pathologists. Davey and coworkers compared concurrent TPPT slides run through the TIS with conventional smears taken simultaneously from the same women [4]. They found a higher ASC-US rate in the slides screened with TIS assistance but the differences between TPPT and conventional cytology confound the results because liquid-based testing itself may have influenced the ASC-US rate relative to smeared slides independent of the effect of the TIS.

Another significant difference between our study and those performed previously was our use of reflex HPV test results as a quality metric in our laboratory. We were consciously trying to remain near the benchmark of 50% HR-HPV positive ASC-US interpretations established by the ALTS trial [11]. This probably contributed to our result of essentially equivalent ASC-US rates in the TIS and MS cases. Our data support the conclusion that by applying this metric the ASC-US rate can be maintained at a stable level regardless of screening method.

Some criticisms of the ALTS trial have been put forward. The patients used in the trial were younger and at higher risk for HPV exposure than the population at large [16], raising the possibility that the results may not be universally applicable. Furthermore, unlike some trials [17,18], not all enrolled women underwent standardized colposcopy and biopsy, leading to verification bias. While we acknowledge these study weaknesses, as the only large prospective trial performed in the United States, the ALTS trial is nevertheless the best available benchmark. Our study population demographics resemble those of the ALTS trial and our clinical follow-up also has a verification bias derived from the same cause, leading us to accept the ALTS trial as a good model for our practice. However, striving for a 50% HR-HPV positive rate in ASC-US in accord with the ALTS trial, as we have done, is not a necessary part of employing HPV data to control ASC-US rates when introducing the TIS. Maintaining HR-HPV positive rates at whatever level a given laboratory deems to be appropriate should have equivalent utility for this purpose.

Ideally any new technology would improve both the sensitivity and the specificity of the Pap test. Although studies agree that the TIS improves sensitivity, the effect on specificity is far less clear. In the current environment, with discussions beginning as to whether HPV testing should replace cytology as the preferred mode of screening [19], it is important to remember that the primary advantage of Pap testing lies in its superior specificity. Any substantial increase in the ASC-US rate attributable to the TIS would almost certainly decrease the specificity of the test, undermining its preferability as a screening tool. We have demonstrated that the TIS does not necessarily alter the ASC-US rate. Thus we believe it is possible to enjoy the benefits of the TIS without deleterious effects on the specificity of the Pap test.

## Conclusion

Our results indicate that use of the TIS has had no appreciable effect on the ASC-US rate in our laboratory. The percentage of TPPT interpreted as ASC-US was essentially the same in cases screened by the TIS as in TPPT screened by MS. This indicates that the use of the TIS, with its productivity benefits, appears not to be having a significant negative impact on the performance characteristics of the Pap test. We believe that the use of the HR-HPV positivity rate in ASC-US as a performance metric contributed to our ability to maintain a stable ASC-US rate while using the TIS.

## List of abbreviations

ASC-US – atypical squamous cells of undetermined significance; CIN – cervical intraepithelial neoplasia; CT – cytotechnologist; FOV – fields of view; HPV – Human Papillomavirus; HR – high-risk; HSIL – high-grade squamous intraepithelial lesion; LSIL – low-grade squamous intraepithelial lesion; MS – manual screening; Pap test – Papanicolaou test; SIL – squamous intraepithelial lesion; TIS – ThinPrep^® ^Imaging System; TPPT – ThinPrep^® ^Papanicolaou test.

## Competing interests

The authors declare that they have no competing interests.

## Authors' contributions

MT collected and compiled the study data, performed the statistical analyses, and drafted the manuscript. DR assisted in the design of the study and contributed to data collection. TB assisted in the design of the study and helped to revise the manuscript. RH supervised the study and helped to revise the manuscript. All authors read and approved the final manuscript.
